# Evaluating the contribution of a scaled up community-based overweight prevention programme in the Netherlands to children’s health behaviours and BMIz

**DOI:** 10.1186/s12966-025-01784-x

**Published:** 2025-06-18

**Authors:** Irma Huiberts, Ehsan Motazedi, Famke J. M. Mölenberg, Amika S. Singh, Dorine Collard, Mai J. M. ChinAPaw, Frank J. van Lenthe

**Affiliations:** 1 Amsterdam UMC, Location Vrije Universiteit Amsterdam, Public and Occupational Health, De Boelelaan 1117, Amsterdam, The Netherlands; 2 Mulier Instituut, Utrecht, The Netherlands; 3 Health Behaviors & Chronic Diseases and Methodology, Amsterdam Public Health, Amsterdam, The Netherlands; 4 Department of Public Health, Erasmus MC University Medical Center Rotterdam, Rotterdam, The Netherlands; 5 School and Sport, Human Movement, Applied University of Windesheim, Zwolle, The Netherlands

**Keywords:** Community-based, Child, Obesity, Overweight, Prevention, Difference-in-differences

## Abstract

**Background:**

Community-based overweight prevention programmes are widely implemented, however, little is known about their effectiveness after scale-up. This study examines the contribution of a scaled up community-based overweight prevention programme in the Netherlands: Healthy Youth, Healthy Future (JOGG) to children’s BMIz, adherence to fruit and/or vegetable consumption guidelines, and minutes of moderate to vigorous physical activity (MVPA) per week.

**Methods:**

In this observational study we used repeated cross-sectional data from 5 to 11- and 12-18-year-old participants in the annual Dutch national health survey (2006–2019) and applied two analytical methods for more robust inference. First, we applied linear mixed models to assess the association between JOGG exposure for at least 18 months (*n* = 1,008) vs. no exposure (*n* = 23,779) and the outcomes and assessed whether this association differed across subgroups defined by age, socioeconomic position (SEP), or migration background. Second, we compared outcome trends in JOGG and non-JOGG municipalities before and after implementation, using a difference-in-differences approach, to account for unobserved time-invariant confounders.

**Results:**

Results showed no significant association between JOGG exposure and BMIz or MVPA. However, JOGG exposure was associated with higher adherence to fruit and/or vegetable consumption guidelines in 12- to 18-year-olds (log odds 1.82, 95%CI 0.23, 3.41). We observed no differential associations across subgroups and no differences in outcome trends between JOGG and non-JOGG municipalities.

**Conclusions:**

The scale up of JOGG in the Netherlands between 2010 and 2019 potentially contributed to higher fruit and vegetable consumption but not to BMIz or MVPA levels. Further examination of the implementation process may provide insight in underlying mechanisms and contribution of JOGG.

**Supplementary Information:**

The online version contains supplementary material available at 10.1186/s12966-025-01784-x.

## Background

Childhood overweight and obesity (hereafter referred to as *overweight*) are a worldwide and growing health problem [1] with significant consequences for children’s physical and mental health as well as for population health and economies [2]. The growing prevalence of childhood overweight is a complex problem driven by multiple interacting economic, social, physical, and political factors [3–5]. Overweight levels are not equally distributed across the population; they tend to be higher among children in families with a lower socioeconomic position (SEP) [1, 6].

Due to its complex aetiology, childhood overweight prevention requires a holistic and sustainable approach [7]. Community-based overweight prevention programmes, which target multiple drivers of unhealthy behaviour in children’s living environment with a community-wide approach [5, 7], have therefore been suggested as promising [7–9]. A key strategy in community-based overweight prevention programmes is to build community capacity for overweight prevention by raising awareness and ownership for this problem in the community and by providing community stakeholders with the support, skills and resources needed to develop solutions and take collective action [10–12]. Thereby, contributing to sustainable changes in the community that promote healthy behaviours.

Various community-based overweight prevention programmes have been developed and found effective in decreasing overweight prevalence [13–18] and in promoting health equity [19, 20] in intervention communities. Consequently, promising programmes have been scaled up and widely implemented [21]; however, data are limited regarding sustainability and the long-term effect of these programmes after scale-up [22–26]. Additionally, it remains unknown whether scaled-up community-based overweight prevention programmes affect existing inequalities in overweight levels [26, 27] and there are concerns that some programmes may widen inequalities when socioeconomically advantaged groups benefit most from them [27].

We evaluated the effect of the scaled-up implementation of a community-based overweight prevention programme in the Netherlands: the Healthy Youth, Healthy Future (JOGG) approach [28, 29]. The JOGG approach was initially based on the successful French EPODE programme [30–32] and was first implemented in 2010 in five Dutch municipalities. By 2023, the JOGG approach had been scaled up to 210 municipalities, over two thirds of municipalities in the Netherlands. Previous studies examining the JOGG approach’s effect on health behaviours and overweight in children in the Netherlands have reported mixed results. Kobes et al. [33] analysed health services registration data and observed a decline in overweight prevalence among children in low SEP areas following long-term implementation of JOGG. In contrast, Blokstra et al. [34, 35], using national health survey data, found no significant differences in overweight rates between matched groups of children living in JOGG versus non-JOGG areas. Beyond limitations related to the secondary data sources, all studies report methodological challenges due to non-randomisation and potential selection bias [33–35]. Moreover, the JOGG’s contribution to reducing inequalities has not been examined.

The JOGG approach has been scaled up by professionals and policymakers; consequently, there has been limited control over implementation, which poses challenges for determining the association between the programme and its outcomes. Once they are scaled up, community-based overweight prevention programmes are therefore best evaluated using study designs specifically suited to assess the contribution of interventions in which exposure and implementation are not controlled by researchers [36]– i.e. natural experiment designs [37, 38]. By combining multiple methods that address different sources of bias, these designs strive for the optimal estimate of the programme’s contribution [37].

In this study, we examine the JOGG approach’s contribution to the BMI and health behaviours of children aged 5 to 18 years. Additionally, we explore whether JOGG reduces inequalities by examining differential effects among subgroups targeted by the JOGG approach, which are defined by SEP and migration background. To support robust inference [37, 39], we applied two approaches to assess the JOGG contribution: first, we estimated the expected difference in the outcomes of individual children who had been exposed to JOGG for at least 18 months compared to non-exposed children. Second, we used a difference-in-differences (DID) approach to examine trends in population average outcome levels over time among children living in municipalities that implemented JOGG compared to children in non-JOGG municipalities.

## Methods

This observational study was conducted and reported following the criteria outlined in the Strengthening the Reporting of Observational studies in Epidemiology [40] (additional file 1).

### The JOGG approach

The JOGG approach aims at promoting a healthy lifestyle and preventing childhood overweight by creating a health-promoting social and physical environment in different settings where children live, play, and learn (e.g., schools, neighbourhoods, sports clubs). Within a municipality, a local JOGG team builds community capacity with local stakeholders and stimulates them to collectively implement sustainable actions and policy changes within their organisations [28]. Outputs vary between municipalities and neighbourhoods; they include, for example, health campaigns and activities, local lifestyle programmes, healthier sport canteens and school lunches, and childcare policies [29, 41]. To illustrate, we present examples of two JOGG municipalities in Table 1.

The national JOGG organisation– financed by the Dutch Ministry of Public Health, Welfare and Sport– supports local teams with training, materials, and advice. Teams are encouraged to target specific subgroups with a higher risk of overweight, including children from deprived areas, a lower SEP, and a migration background.

**Table 1:** Examples of JOGG implementation and outputs in two municipalities

**Example 1 ** This municipality initiated JOGG in 2013. A local coordinator was allocated 20 h per week for implementation. The municipality developed a broad JOGG network comprising both public and private partners who participated in annual network meetings and received regular progress updates via newsletters. As a result, many JOGG-related activities took the form of small-scale initiatives led by local partners. For instance, local restaurants organised healthy cooking workshops for schools. The JOGG team continued to lead several key initiatives, including an annual kids’ run, walking events for young children, and school-based campaigns promoting healthy lunchboxes. In addition to these periodic activities, several additional sustainable changes were implemented by local partners. Twenty public water stations were installed at strategic locations, such as parks and school areas. Five sports clubs offered healthy food options in their canteens after support from JOGG. Furthermore, five schools—representing approximately 10% of all schools in the municipality—earned the “Healthy School” certificate by implementing healthier canteens and integrating annual lesson plans on nutrition and healthy lifestyles. At the policy level, the municipality introduced a regulation ensuring that each school was assigned a certified physical education teacher.
**Example 2** This municipality launched JOGG in 2014. A local coordinator was assigned 28 h per week for programme implementation. During the initial years, several initiatives were developed and were later integrated into routine practice. These included a targeted intervention for children with overweight and a school-based physical activity curriculum. Additionally, a day care programme was introduced to promote physical activity and improve motor skills among young children. Staff at all day care centres in the municipality were able to attend free annual training to implement this programme effectively. Over time, 19 primary schools (30% of all primary schools in the municipality) were supported in obtaining the “Healthy School” certificate. Most day care locations in the municipality introduced a healthy nutrition policy. All sporting events organised by the municipal sports service started offering solely water and fruit as refreshments. Over the next years, the municipality plans to expand its focus to include adolescents in secondary schools and to strengthen support for schools in low-SEP neighbourhoods to develop and implement comprehensive health policies.

### Data

We used repeated cross-sectional data from the Dutch national health survey [42] from 2006 to 2019 to assess the contribution of JOGG on children’s BMI z-scores and health behaviours over time. The survey is conducted annually to follow health and lifestyle developments among the Dutch population; approximately 15,000 people of all ages from across the country are invited to participate, and between 60% and 65% typically respond. We chose not to include data after 2019 because the COVID-19 pandemic impacted programme delivery, data collection processes, and trends in health behaviours in the years that followed.

The survey questions for respondents under the age of 12 were answered by a parent or caretaker. Youth aged 12 years and older answered the survey questions themselves. Survey questions for the measures included in this study and the formulation of these questions were consistent across all study years.

Before 2010, the health survey was conducted as a face-to-face interview with an additional paper questionnaire. Since then, respondents have been asked to participate via internet, and those not responding via internet have participated through face-to-face interviews. This change in methodology did not lead to significant breaks in the trends on items included in the current study [43].

### Study sample

**Fig. 1:**
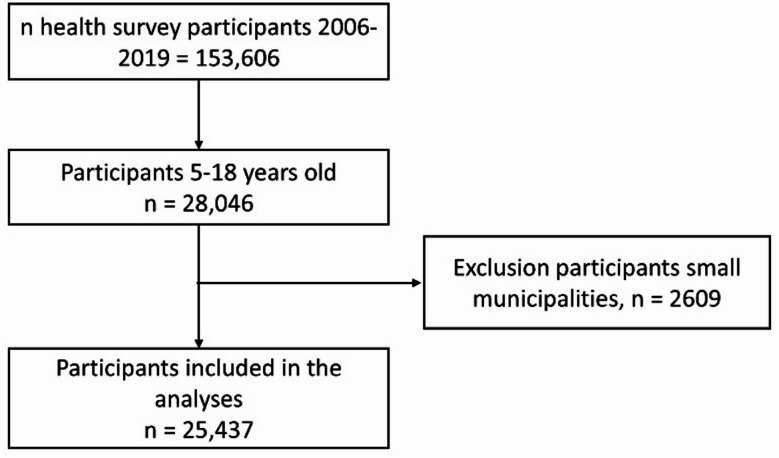
Study participants included in the analyses

Figure 1 illustrates our selection process for participants included in the analyses. We selected participants from 2006, 5 years before the first municipalities started implementing JOGG, to 2019, and we selected those who were 5 to 18 years of age during that period. We excluded people from municipalities with less than 40 participants between 2006 and 2019.

#### JOGG municipalities and exposure

The JOGG approach is usually adopted by a municipality; a local government responsible for a population varying from 10.000 to 150.000 residents. JOGG is then implemented in one or more neighbourhoods in the municipality. Neighbourhoods where JOGG is implemented are often deprived areas. The national JOGG organisation provided information on the municipalities and neighbourhoods where JOGG was implemented (i.e., JOGG neighbourhood) between 2006 and 2019. As a neighbourhood’s exact start month of the JOGG approach was often unclear, we considered them a JOGG neighbourhood from the first of January after the officially administered start year. The number of municipalities implementing the JOGG approach increased each year following its introduction in 2010. By 2019, approximately one-quarter of all Dutch municipalities (*n* = 355) had implemented JOGG.

We defined participant’s association with the JOGG approach in two ways. First, we examined whether participants lived in a JOGG neighbourhood at the year of measurement (*JOGG* = 1) or lived in a non-JOGG neighbourhood, one that had not yet or never implemented the JOGG approach at the year of measurement (*JOGG* = 0). Second, we calculated the number of months that participants had lived in a JOGG neighbourhood after the age of 4, based on current and previous living addresses (using data from Statistics Netherlands). We defined *JOGG exposure* as having lived in JOGG neighbourhoods for at least 18 months (1) [] versus never lived in a JOGG neighbourhood (0). Table 2 provides an overview of the number of JOGG municipalities and neighbourhoods included in the study as well as the number of exposed and unexposed children.

**Table 2:** Number of JOGG municipalities and neighbourhoods in the Netherlands and number of children exposed to the JOGG approach included in the analysis between 2006 and 2019*

	JOGG implementation		Participants		
	Municipalities	Neighbourhoods	JOGG	JOGG exposure > 18 months	No exposure
2006	0	0	0	0	1,698
2007	0	0	0	0	1,502
2008	0	0	0	0	1,693
2009	0	0	0	0	1,558
2010	0	0	0	0	2,882
2011	5	45	70	0	2,384
2012	10	63	91	28	2,253
2013	16	85	111	60	2,128
2014	23	75	92	68	1,489
2015	51	141	178	85	1,388
2016	62	185	223	122	1,254
2017	61	194	228	181	1,333
2018	81	267	307	186	1,162
2019	85	333	392	278	1,055

*The number of JOGG municipalities, neighbourhoods and participants in this table represent those included in the Dutch national health survey. This survey includes a representative sample from the entire Dutch population. Less than a quarter of the Dutch population lived in a JOGG neighbourhood by 2019

### Measures

#### BMIz

Participants were asked to report their weight in whole kilograms (without clothes) and their height in centimetres (without shoes). BMI was calculated as weight (kg) divided by height squared (m^2^) and converted to age- and sex-specific z-scores using the World Health Organization’s reference curves [44]. BMI scores below 10 or above 50 were considered biologically impossible and likely resulting from errors in height or weight; therefore, they were coded as missing values.

#### Physical activity

Physical activity was assessed in children of 12 years and older by the Short QUestionnaire to ASsess Health-enhancing physical activity (SQUASH) [45] in total minutes of moderate to vigorous physical activity (MVPA) per week. The SQUASH includes questions about the average frequency per week and duration per day that participants spend on different commuting, leisure time, and household activities as well as activity at work or school. We followed recommendations for calculations with the SQUASH [46]; accordingly, values of more than 105 hours of total activity per week (over 15 hours per day) were considered unrealistic and coded as missing values.

Information was not available on physical activity for children aged 5 to 11 years.

#### Fruit and vegetable consumption

Since 2014, questions regarding fruit and vegetable consumption have been included. Questions regarding fruit consumption include the number of days per week that children eat fruit and the number of pieces of fruit they eat on those days. For vegetable consumption, children are asked how many days per week they eat vegetables and how many serving spoons they, on average, eat on those days. We calculated whether children adhered to the recommended daily fruit and/or vegetable consumption (0/1) based on the Dutch dietary guidelines for each age group (see [47]).

#### Covariates

The child’s age, sex, migration background, and parental education level were collected via the national health survey. We categorised migration background in line with Statistics Netherlands [48]: [1] Dutch — both the child and parents were born in the Netherlands; [2] Western — the child or one of their parents was born in a country in Europe, North America, or Oceania; [3] non-Western — the child or one of their parents was born in a country in South America, Africa, Asia, or Turkey. We categorised the parental education level (available only for children < 12 years old) from the highest educated parent as low, middle, and high, in line with Statistics Netherlands. Additionally, standardised household income was available from the registry data and was categorised by income quartiles for each participant.

Neighbourhood-level demographic data were available from Statistics Netherlands. The percentage of households at or below the minimum household income in the neighbourhood in each survey year was included as an indicator for neighbourhood deprivation.

### Analysis

All statistical analyses were conducted using STATA version 16. The statistical significance level was set to $\:\alpha\:=0.05$ in all analyses, and 95% CIs were calculated. In each model, children with missing values on the outcome or any of the covariates were not included in the analyses. See additional file 2 for all model formulas.

#### Effect estimate I: JOGG exposure and subgroup differences

We investigated the effect of JOGG exposure (i.e. lived in JOGG neighbourhood for at least 18 months) on children’s BMI z-score and log-transformed MVPA (logPA) using linear mixed models. Similar analyses were performed for the dichotomous variable of adhering to fruit and/or vegetable consumption guidelines using a generalised logistic mixed model. Stratified analyses were performed by age group (5 to 11 and 12 to 18 years old) because the availability of covariates and reporting method (self- or parent-reported) varied between these age groups. Each model was adjusted for age (in years), sex, migration background, parent education level (available only for the 5- to 11-year-old group), household income, neighbourhood deprivation, and survey year. Additionally, models were adjusted for the interaction of JOGG exposure with age because the effect of JOGG may vary across ages and the chance of being exposed to the JOGG approach for at least 18 months was greater for older children. We included random intercepts in all models to account for clustering of the outcomes by municipality. We excluded participants who were exposed between 1 and 17 months from the analysis (*n* = 690).

Moreover, to study the differential effects of JOGG exposure between subgroups, we alternately included the interaction terms between JOGG exposure and migration background, parent education level, and household income in the adjusted models.

#### Effect estimate II: DID

Additionally, we estimated the difference in trends of the average outcome levels over time for the group of participants living in a JOGG neighbourhood (*JOGG =* 1) compared to the group of participants living in a non-JOGG neighbourhood (*JOGG =* 0). This approach, also known as DID [49], examines JOGG’s effects while allowing for structural differences between the JOGG and non-JOGG group, thereby reducing bias from unobserved time-invariant confounders [37, 49]. In the JOGG approach, such confounders might include the possibility that introducing JOGG reflects the presence of more extensive health policies or a response to higher prevalence of health problems in these neighbourhoods or municipalities.

We employed linear and logistic mixed models with an interaction term for *JOGG* and time (2006–2019) to examine whether introducing the JOGG approach affected time trends in outcome levels for (1) BMI z-scores, (2) logPA and (3) adherence to fruit and/or vegetable consumption guidelines. Participants of all ages (5–18 years) were included in the models and we included random intercepts for municipality to account for clustering of outcomes by municipality.

The core assumption in DID is that time trends of the outcome levels in JOGG and non-JOGG neighbourhoods would have been parallel had JOGG not been introduced (parallel trends assumption). To test this assumption, we examined whether time trends in outcome levels were parallel in the period before JOGG was introduced (2006–2010) by visualising the time trends and by using linear and logistic mixed models with an interaction term for living in a future JOGG neighbourhood and time. Potential biases in DID analysis can stem from changes in the composition of the groups over time [49] and from staggered programme introduction when its effects vary over time [50], which may be expected since time is required for outcomes to change after JOGG is introduced. Therefore, we examined whether covariate distributions between the JOGG and non-JOGG groups remained constant over time [49] (additional file 3). Additionally, we conducted stratified analyses for each introduction year, which provide insight into the impact of compositional changes and staggered introductions on the overall effect estimate [50].

## Results

### Descriptives

Table 3 presents the study sample’s characteristics according to JOGG exposure. On average, the exposed group had higher BMI z-scores, lower MVPA levels, and slightly more frequent adherence to fruit and/or vegetable consumption guidelines than the non-exposed group. The exposed and non-exposed groups were similar in sex, age, and parental education. As expected, children who were exposed to the JOGG approach more often had a non-Western migration background and lived in lower income households that were in neighbourhoods with higher deprivation levels. Additional file 3 includes the distribution of these characteristics between future JOGG and non-JOGG municipalities over time. These demonstrate that the number of children with a non-Western migration background from lower income households was structurally higher in JOGG municipalities even before JOGG was introduced, which indicates that the JOGG approach was more often introduced in neighbourhoods where families have lower income and migration backgrounds.

**Fig. 2:**
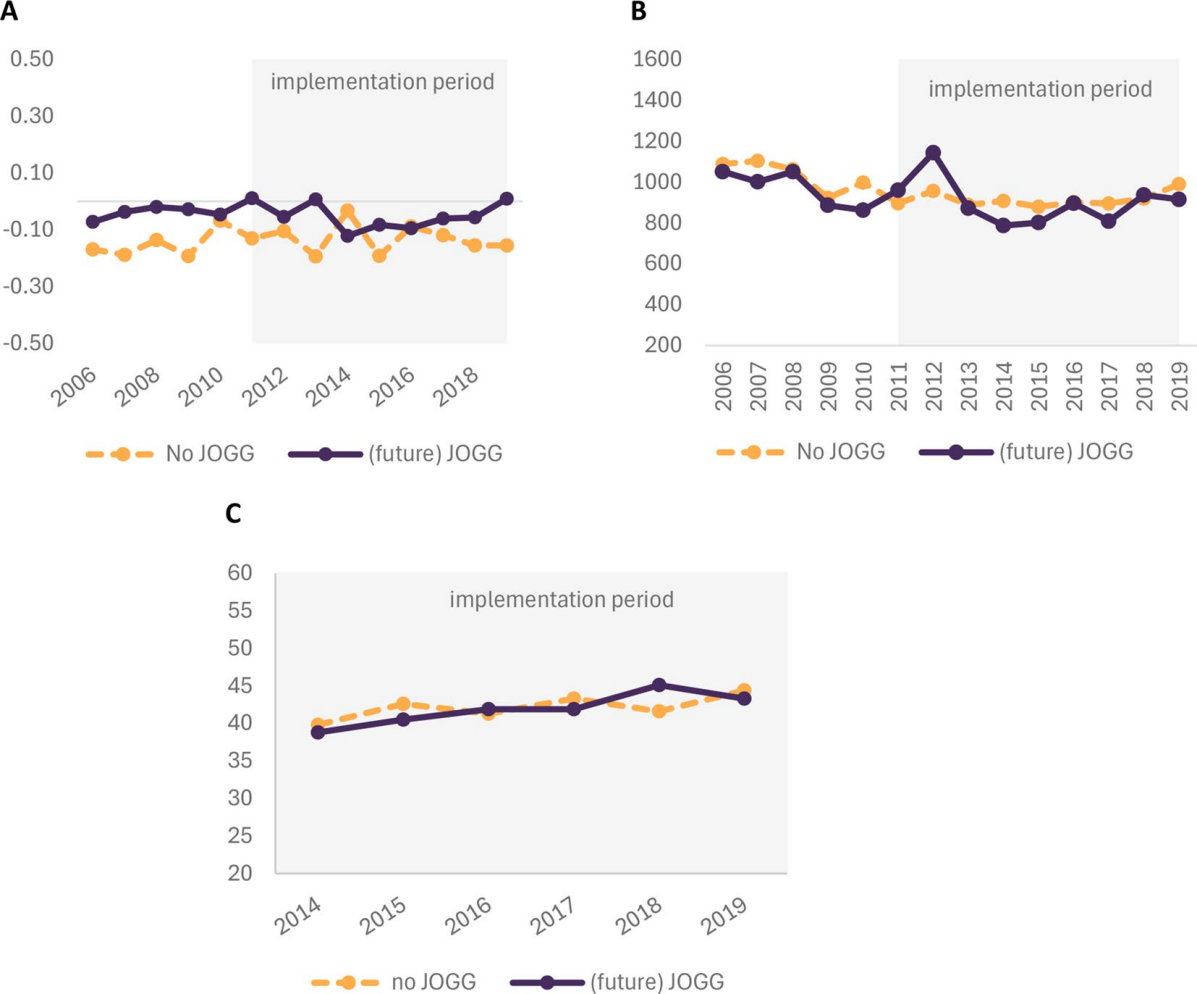
Descriptive outcome trends (2006–2019) for children in municipalities that never implemented JOGG compared to municipalities that implemented JOGG after 2010. **A** Mean BMI z-scores. **B** Mean minutes of MVPA per week. **C** Percentage of children adhering to fruit and/or vegetable consumption guidelines

Figure 2 illustrates the outcome levels for each survey year according to whether children lived in a municipality that did or did not implement JOGG (in the future). The group of children living in JOGG municipalities had slightly higher BMI z-scores than children in non-JOGG municipalities but similar MVPA levels and adherence to fruit and vegetable consumption guidelines. For both groups, MVPA levels declined slightly over time. Adherence to fruit and vegetable consumption guidelines increased slightly over the years.

**Table 3:** Participant characteristics according to JOGG exposure

	Age 5–11 years		Age 12–18 years	
	No JOGG exposure *n* = 12,321	JOGG exposure > 18 months *n* = 490	No JOGG exposure *n* = 11,458	JOGG exposure > 18 months *n* = 518
BMI z-score				
Mean	− 0.12	0.10	− 0.11	0.02
*SD*	1.34	1.28	1.07	1.10
*N* missing	1,710	29	603	12
Minutes of MVPA per week				
Mean	-	-	950	877
*SD*	-	-	847	839
*N* missing	-	-	3,712	34
Adherence to fruit and/or vegetable consumption guidelines*	53% (*n* = 2,087)	56% (*n* = 246)	31% (*n =* 1,156)	36% (*n* = 172)
Sex				
Male	50%	53%	50%	46%
Female	50%	47%	50%	54%
Migration background				
Dutch	79%	57%	79%	58%
Western	7%	7%	7%	9%
Non-Western	14%	37%	14%	33%
Standardised household income				
Unknown	0.7%	-	1%	-
Quartile 1 (lowest)	21%	30%	19%	26%
Quartile 2	25%	25%	22%	26%
Quartile 3	28%	25%	30%	25%
Quartile 4 (highest)	24%	20%	28%	21%
Parental education				
Low	12%	15%	-	-
Middle	33%	32%	-	-
High	42%	45%	-	-
Unknown	14%	9%		
Neighbourhood deprivation: Percentage of minimum income households				
Mean	7.1	10.3	7.0	9.8
*SD*	4.8	7.3	4.7	7.5

* available only from 2014–2019

### Effect estimate I: JOGG exposure and subgroup differences

Table 4 presents the results of the linear mixed-model analysis for the 5- to 11-year-old group. No significant effect of exposure to JOGG was found in BMI z-scores or adherence to fruit and/or vegetable consumption guidelines, and effects did not differ according to migration background, parental education, or household income.

Table 5 provides the results for the 12- to 18-year-old group. No significant effects of JOGG exposure or differential effects across subgroups were found on either BMI z-scores or MVPA (logPA). Youth who were exposed to the JOGG approach at the survey time had significantly higher odds of adhering to fruit and/or vegetable consumption guidelines (log odds 1.82, 95% CI [0.23, 3.41], *p* = 0.03) when age is zero. The beneficial effect of JOGG exposure decreased with age (log odds JOGG x age − 0.12 per year, 95% CI [-0.22, − 0.01], *p* = 0.03). To facilitate the interpretation of these results we calculated the Risk Difference (RD) between the exposed and non-exposed group, according to age (additional file 4). The probability of adhering to fruit and/or vegetable guidelines for 12 year olds was 9.8% higher in the exposed group than in the non-exposed group (RD 0.098, CI [0.01], 0.18, *p* = 0.03). This difference in probabilities diminished with age, reaching zero by age of 15.

**Table 4:** Effects of JOGG exposure (> 18 months) on BMI z-scores and adherence to fruit and/or vegetable consumption guidelines in 5- to 11-year-old group

		BMIz *n =* 10,941			Adherence to fruit and/or vegetable consumption guidelines *n =* 4,305		
		ICC^A^	Estimate (95% CI)	*p*	ICC^A^	Estimate in log odds^B^ (95% CI)	*p*
**0**	*Random effect of municipality*	0.01	-	-	0.03	-	-
**1**	**JOGG exposure**	0.00	− 0.06 (-0.60, 0.47)	0.81	0.01	− 0.58 (-1.50, 0.34)	0.21
	**JOGG exposure x age (in years)**		0.02 (-0.04, 0.08)	0.64		0.05 (-0.05, 0.16)	0.32
**2**	**JOGG exposure x migration background**	0.00			0.01		
	Dutch (ref)						
	Western		− 0.14 (-0.65, 0.37)	0.59		− 0.54 (-1.38, 0.30)	0.21
	Non-Western		− 0.05 (-0.32, 0.22)	0.73		− 0.21 (-0.69, 0.27)	0.39
**3**	**JOGG exposure x parent education level**	0.00			0.01		
	Low (ref)						
	Middle		− 0.22 (-0.61, 0.17)	0.27		− 0.35 (-1.01, 0.31)	0.29
	High		− 0.07 (-0.44, 0.29)	0.69		− 0.43 (-1.06, 0.21)	0.19
	Unknown		0.16 (-0.41, 0.74)	0.57		− 0.32 (-1.46, 0.81)	0.58
**4**	**JOGG exposure x household income**	0.00			0.01		
	Quartile 1 (lowest, ref)						
	Quartile 2		0.01 (-0.33, 0.35)	0.97		0.07 (-0.52, 0.65)	0.82
	Quartile 3		0.23 (-0.11, 0.57)	0.18		− 0.17 (-0.75, 0.40)	0.55
	Quartile 4 (highest)		0.17 (-0.18, 0.53)	0.34		0.08 (-0.54, 0.70)	0.81

Models 1–4 were adjusted for age, JOGG exposure x age interaction, sex, migration background, parental education, parental income, neighbourhood deprivation, survey year, and random effect of municipality

*Significant at α = 0.05

^A^ Intraclass correlation coefficient: indicates the proportion of variability in the outcome that is attributable to municipality

^B^ Log odds < 0 indicate a negative effect on the outcome; > 0 indicate a positive effect on the outcome

**Table 5:** Effects of JOGG exposure (> 18 months) on BMI z-scores, MVPA, and adherence to fruit and/or vegetable consumption guidelines in 12- to 18-year-old group

		BMIz *n =* 11,233			MVPA (logPA) *n =* 7,985			Adherence to fruit and/or vegetable consumption guidelines *n =* 4,213		
		ICC^A^	Estimate (95% CI)	*p*	ICC^A^	Estimate (95% CI)	*p*	ICC^A^	Log odds^B^	*p*
**0**	*Random effect of municipality*	0.00			0.01			0.02		
**1**	**JOGG exposure**	0.00	0.45 (-0.27, 1.18)	0.22	0.00	− 0.09 (-0.71, 0.53)	0.78	0.01	1.82 (0.23, 3.41)	0.03*
	**JOGG exposure x age (in years)**		− 0.03 (-0.08, 0.02)	0.22		0.00 (-0.04, 0.05)	0.83		− 0.12 (-0.22, − 0.01)	0.03*
**2**	**JOGG exposure x migration background**	0.00			0.00			0.01		
	Dutch (ref)									
	Western		0.35 (-0.01, 0.70)	0.06		0.11 (-0.20, 0.42)	0.50		− 0.08 (-0.87, 0.71)	0.84
	Non-Western		− 0.06 (-0.27, 0.15)	0.58		− 0.09 (-0.27, 0.09)	0.34		− 0.19 (-0.66, 0.28)	0.43
**3**	**JOGG exposure x household income**	0.00			0.00			0.01		
	Quartile 1 (lowest, ref)									
	Quartile 2		0.11 (-0.15, 0.38)	0.41		0.06 (-0.17, 0.29)	0.62		0.18 (-0.40, 0.76)	0.55
	Quartile 3		0.04 (-0.23, 0.30)	0.79		0.00 (-0.23, 0.22)	1.00		− 0.15 (-0.74, 0.44)	0.62
	Quartile 4 (highest)		− 0.07 (-0.35, 0.21)	0.61		− 0.03 (0.27, 0.20)	0.78		0.26 (-0.35, 0.86)	0.41

All models were adjusted for age, JOGG exposure x age interaction, sex, migration background, parental income, neighbourhood deprivation, survey year, and random effect of municipality

*Significant effect at α = 0.05

^A^ Intraclass correlation coefficient: indicates the proportion of variability in the outcome that is attributable to municipality

^B^ Log odds < 0 indicate a negative effect on the outcome; > 0 indicate a positive effect on the outcome

### Effect estimate II: DID

There were no significant differences in children living in JOGG neighbourhoods compared to children who lived in a neighbourhood where JOGG was never implemented in BMI z-scores or logPA trends in the pre-implementation period (2006–2010), which supports the parallel trends assumption. Because adherence to fruit and vegetable consumption guidelines has been measured only since 2014, we could not examine outcome trends in the pre-implementation period.

Table 6 presents the results of the DID analysis. Outcome trends in BMI z-scores and MVPA levels were not significantly different between children living in JOGG vs. non-JOGG neighbourhoods between 2006 and 2019. Time trends for adherence to fruit and/or vegetable consumption guidelines were not significantly different between children living in JOGG or non-JOGG neighbourhoods between 2014 and 2019. Results of the stratified analysis for each JOGG introduction year consistently exhibited no JOGG impact on outcome trends (additional file 5). Overall, the results indicated that JOGG implementation did not have a significant effect on BMI z-scores, MVPA levels, or adherence to fruit and/or vegetable consumption guidelines.

**Table 6:** Estimates for the difference in outcome trends between JOGG and non-JOGG municipalities

	Outcome	Interaction parameter estimates JOGG x time	
		**Beta (95% CI)**	***p*** **-value**
1)	BMIz (2006–2019)	− 0.02 (-0.05, 0.01)	0.13
2)	MVPA (logPA; 2006–2019)	− 0.02 (-0.01, 0.05)	0.22
		**Log odds** ^A^ **(95% CI)**	***p*** **-value**
3)	Adherence to fruit and/or vegetable consumption guidelines (2014–2019)	0.01 (-0.06, − 0.08)	0.75

All models were adjusted for the random effect of municipality

(1) *n =* 23,028, (2) *n =* 8,342, (3) *n =* 9,065

^A^ Log odds < 0 indicate a negative effect on the outcome; > 0 indicate a positive effect on the outcome

## Discussion

Implementing the JOGG approach, a community-based overweight prevention programme for children and youth in the Netherlands, did not significantly affect children’s BMI z-scores or MVPA. We observed that youth 12 to 18 years of age who were exposed to the JOGG approach were slightly more likely to adhere to fruit and/or vegetable consumption guidelines than those who were not exposed. However, this benefit decreased with age and this finding could not be confirmed by comparing average outcome levels over time among children living in JOGG municipalities to children in non-JOGG municipalities. We found no evidence for the differential effects of JOGG exposure across subgroups defined by SEP and migration background. Therefore, the JOGG approach appears not to widen or reduce inequalities in BMI or health behaviours.

Our findings are inconsistent with those of previous studies on community-based overweight prevention programmes that were not scaled up, which predominantly suggest that these programmes can reduce overweight prevalence [13–18]. This inconsistency may be due to differences in each municipality’s JOGG implementation. Programmes that were previously effective entailed a controlled, strategic, and intense combination of interventions and policy changes within one or a few communities (e.g., [51, 52]). The JOGG approach, however, is implemented by local municipality teams that vary significantly in, for example, resources and local stakeholder attitudes towards the programme. Consequently, implementation differs widely [41, 53, 54], with some forms being more intense or effective than others.

Moreover, isolating JOGG effects from the broader context, especially from other policy measures within municipal health promotion [23], is nearly impossible. Most municipalities in the Netherlands implement some programmes that promote physical activity [55] regardless of their JOGG status. Consequently, the contrast between JOGG and non-JOGG municipalities regarding physical activity promotion may be minimal, thereby potentially explaining the lack of a JOGG effect on MVPA. However, fruit and vegetable consumption is not typically addressed in municipal or school policies [56]. This issue may receive more attention in JOGG municipalities, which could account for the observed small contribution of JOGG to fruit and vegetable consumption.

Previous researchers have argued that a solid process evaluation could consider differences in implementation and context [57, 58]. However, selecting clear-cut indicators of successful implementation is difficult due to the complexity of the causal pathway from the implementation of a community-based overweight prevention programme to health outcomes [28, 57] because the programme’s components interact with the context and each other [59], thus complicating the attribution of effects solely to JOGG. Additionally, the combinations of components that lead to success may vary between contexts [23, 59]. Therefore, we recommend further studies on the intermediate outcomes (e.g., policies and practices) [57, 59] and the mechanisms through which the programme contributes to these outcomes [26, 28, 59]. This may improve understanding of why and in what context the programme is effective [25, 28] while also strengthening causal inferences [57]. Evidence on the characteristics of community-based overweight prevention programmes that contribute to success is essential for policymakers to successfully plan and implement these programmes within their specific context [26] and to achieve meaningful impact on overweight prevention.

We should note that it may be unrealistic for community-based prevention programmes alone to reduce the prevalence of overweight in the long term, because of the complex system of multiple factors that drive this problem [3–5]. JOGG focusses on sustainable changes that are systematically embedded into an organisation’s attitude, knowledge, and policies and solidified in infrastructure (e.g., playgrounds) [28]. There is evidence that such measures contribute to health outcomes [2] and are sustainable in the long term [60]; however, such changes alone are insufficient to reduce the prevalence of overweight, as prevention eventually requires broad and sustainable system change that is driven by community-based action as well as top-down national policy action from public and private sectors [61]. This suggests that in decision making on the adoption of community-based programmes it is essential to consider the contribution of such programmes to a broader overweight prevention strategy, rather than solely focusing on individual health outcomes [62, 63]. For example, by evaluating sustainable changes to the community system that result from the programme.

### Strengths and limitations

A strength of this study is the application of two effect estimates. Estimations of (1) the effects of exposure and (2) the difference in time trends between JOGG and non-JOGG municipalities rely on different assumptions and effect definitions; consequently, limitations in either one can be addressed by the advantages of the other [37, 39]. By applying Estimate 1, we controlled for several observed confounders and examined subgroup differences. However, structural differences between JOGG and non-JOGG groups may have caused bias. Examining the difference in time trends (Estimate 2) allowed for structural differences between the groups, thereby reducing bias from unobserved time-invariant confounders. In both analyses, we considered municipality-level clustering to avoid bias due to municipal differences [36] but neighbourhood-level bias may remain.

Nevertheless, there may still be remaining sources of bias. Neither of the two methods employed could adjust for unobserved confounders that differentially affected the exposed and unexposed groups and outcomes over time [37, 49]; furthermore, the small sample size prevented our ability to account for neighbourhood-level clustering. Adherence to fruit and/or vegetable consumption guidelines was available only from 2014 onwards, so we could not examine outcome trends before JOGG implementation. Consequently, it remains unclear whether JOGG’s small positive effect on adherence to fruit and/or vegetable consumption guidelines in the first analysis might have been biased by differences between the JOGG and non-JOGG groups before JOGG implementation.

Moreover, there are some limitations to the data. We used data only until 2019, although many more municipalities introduced JOGG after this time. The study sample may have been insufficient to detect results since effect sizes of community-based overweight prevention programmes are usually small [8]. Additionally, height and weight was self- or parent-reported and may have suffered from social desirability [57]. Finally, data were not available on other possible relevant outcomes, such as the consumption of water and sugar-sweetened beverages.

Lastly, the limited information available on the diverse ways in which the JOGG approach has been implemented represents a limitation of this study. As a result of this variability, some children in our sample may have had limited exposure to the activities and environmental changes associated with JOGG. For instance, adolescents aged 12–18 may reside in municipalities where the JOGG approach primarily targets younger children within childcare settings. This heterogeneity in exposure may reduce the likelihood of detecting an effect of the JOGG approach at the individual level.

Despite these limitations, the current approach is valuable in evaluating the JOGG approach. A Randomized Controlled Trial is often suggested as the gold standard for evaluating health interventions; however, the core limitation of this approach is that the randomisation that is used to reduce bias is often not possible when studying long-term outcomes after scale-up and can result in evidence that no longer reflects the reality of practice [38]. Considering the conditions in which the programme is implemented, including local policies and differences in implementation, provides a more realistic and generalisable estimate of the programme’s contributions in a population than a controlled setting [24, 38] and may therefore be more informative for policymakers.

## Conclusions

We studied the contribution of a nationally scaled-up community-based overweight prevention programme in the Netherlands to children BMIz scores, MVPA and fruit and vegetable consumption between 2010 and 2019. Although, no significant differences in BMI z-scores or MVPA levels were found, in 12–18 year olds the probability of adhering to fruit and/or vegetable guidelines was higher in the group that had been exposed to the JOGG approach than in the non-exposed group. Examination of the implementation process and intermediate outcomes using both quantitative and qualitive methods is needed to unravel the working mechanisms and effectiveness of community-based overweight prevention programmes after scale-up. This will provide policymakers with more relevant information to decide on the adoption of these programmes and maximise their implementation and impact on health outcomes.

## Electronic supplementary material

Below is the link to the electronic supplementary material.

Supplementary Material 1: STROBE checklist.

Supplementary Material 2: Model formulas.

Supplementary Material 3: Distribution of covariates in non-JOGG and (future) JOGG group over time.

Supplementary Material 4: Risk Differences.

Supplementary Material 5: stratified analysis for different introduction years.

## Data Availability

Data is available from Statistics Netherlands.

## Abbreviations

JOGG: Healthy Youth, Healthy Future
MVPA: Moderate to vigorous physical activity
SEP: Socioeconomic position
BMI: Body Mass Index
DID: Difference-in-differences
logPA: Log-transformed MVPA
CI: Confidence interval
RD: Risk difference
