# Supplementary material for: Evaluating the contribution of a scaled up community-based overweight prevention programme in the Netherlands to children’s health behaviours and BMIz
Source: Int J Behav Nutr Phys Act. 2025 Jun 18;22:79. doi: 10.1186/s12966-025-01784-x (PMC12177978; doi:10.1186/s12966-025-01784-x)
Supplement: Supplementary file 2 — Supplementary Material 2: Model formulas. [file 12966_2025_1784_MOESM2_ESM.pdf]

## Additional file 2 model formulas

### Effect estimate I

*Children 5-12 years old*

Using linear mixed models, we modelled the effect of exposure to the JOGG approach on BMIz, according to:

$$y_{ij} = \beta_0 + b_{0j} + \beta_1 exposure_{ij} + \beta_2 age_{ij} + \beta_3 sex_{ij} + \beta_4 migrationbackground_{ij} + \beta_5 parenteducationlevel_{ij} + \beta_6 householdincome_{ij} + \beta_7 neighbourhooddeprivation_{ij} + \beta_8 surveyyear_{ij} + \beta_9 exposure * age_{ij} + \in_{ij} \quad 1$$

Where $\beta_1 exposure = 1$ if the individual lived in a neighbourhood where JOGG was implemented for at least 18 months (otherwise $\beta_1 exposure = 0$), $b_{0j}$ is the random intercept of the outcome for a municipality $j$, and $\in_{ij}$ is the model residual.

In order to study differential effects of JOGG exposure between subgroups, we alternately included the interaction terms between JOGG exposure and migration background, parent education level and household income in the model, specified as follows:

$$y_{ij} = \beta_0 + b_{0j} + \beta_1 exposure_{ij} + \beta_2 age_{ij} + \beta_3 sex_{ij} + \beta_4 migration\_background_{ij} + \beta_5 parent\_education\_level_{ij} + \beta_6 household\_income_{ij} + \beta_7 neighbourhooddeprivation_{ij} + \beta_8 survey\_year_{ij} + \beta_9 exposure * age_{ij} + \beta_{10} exposure * migrationbackground_{ij} + \in_{ij} \quad 2$$

$$y_{ij} = \beta_0 + b_{0j} + \beta_1 exposure_{ij} + \beta_2 age_{ij} + \beta_3 sex_{ij} + \beta_4 migrationbackground_{ij} + \beta_5 parenteducationlevel_{ij} + \beta_6 householdincome_{ij} + \beta_7 neighbourhooddeprivation_{ij} + \beta_8 surveyyear_{ij} + \beta_9 exposure * age_{ij} + \beta_{10} exposure * parenteducationlevel_{ij} + \in_{ij} \quad 3$$

$$y_{ij} = \beta_0 + b_{0j} + \beta_1 exposure_{ij} + \beta_2 age_{ij} + \beta_3 sex_{ij} + \beta_4 migrationbackground_{ij} + \beta_5 parenteducationlevel_{ij} + \beta_6 householdincome_{ij} + \beta_7 neighbourhooddeprivation_{ij} + \beta_8 surveyyear_{ij} + \beta_9 exposure * age_{ij} + \beta_{10} exposure * householdincome_{ij} + \in_{ij} \quad 4$$

Similar analyses were performed using generalized logistic mixed models with the logit link to estimate the effect of exposure on adherence to fruit/vegetable guidelines over the time.

*Youth 12-18 years old*

Model specifications were the same for youth 12-18 years old, except that the covariate parental education level was not available. We modelled the effect of exposure to the JOGG approach on logPA and BMIz using linear mixed models, according to:

$$y_{ij} = \beta_0 + b_{0j} + \beta_1 exposure_{ij} + \beta_2 age_{ij} + \beta_3 sex_{ij} + \beta_4 migrationbackground_{ij} + \beta_5 householdincome_{ij} + \beta_6 neighbourhooddeprivation_{ij} + \beta_7 surveyyear_{ij} + \beta_8 exposure * age_{ij} + \in_{ij} \quad 1$$

$$y_{ij} = \beta_0 + b_{0j} + \beta_1 exposure_{ij} + \beta_2 age_{ij} + \beta_3 sex_{ij} + \beta_4 migrationbackground_{ij} + \beta_5 householdincome_{ij} + \beta_6 neighbourhooddeprivation_{ij} + \beta_7 surveyyear_{ij} + \beta_8 exposure * age_{ij} + \beta_9 exposure * migrationbackground_{ij} + \in_{ij} \quad 2$$

$$y_{ij} = \beta_0 + b_{0j} + \beta_1 exposure_{ij} + \beta_2 age_{ij} + \beta_3 sex_{ij} + \beta_4 migrationbackground_{ij} + \beta_5 householdincome_{ij} + \beta_6 neighbourhooddeprivation_{ij} + \beta_7 surveyyear_{ij} + + \beta_8 exposure * age_{ij} + \beta_9 exposure * householdincome_{ij} + \in_{ij} \quad 3$$

**Effect estimate II**

Using linear mixed models, we modelled the outcomes BMIz and logPA for individual $i$ in municipality $j$ at the measurement year $t$ (from 2006 to 2019) $y_{ijt}$. We estimated the effects of the outcome measurement year, $\beta_1 time$, the effect of JOGG implementation in the municipality $j$ in which individual $i$ lives at the measurement year $t$, $\beta_2 JOGG_{ijt}$, and the interaction of the measurement year with the participation in JOGG , $\beta_3 time * JOGG_{ijt}$, according to:

$$y_{ijt} = \beta_0 + b_{0j} + \beta_1 time + \beta_2 JOGG_{ijt} + \beta_3 time * JOGG_{ijt} + \epsilon_{ij}$$

Where $\beta_1 time$ is the outcome measurement year, $JOGG_{ijt} = 1$ if municipality $j$ where individual $i$ lives, participates in JOGG at measurement year $t$ and afterwards, (otherwise $JOGG_{ijt} = 0$), $b_{0j}$ is the random intercept of the outcome for a municipality $j$ and $\epsilon_{ijt}$ is the model residual. The coefficient $\beta_3$ is the interaction term between time and JOGG participation and depicts the intervention effect on the outcome.

We tested the parallel trends assumption by comparing the trend of the outcomes before the introduction of JOGG, i.e. with $t$ between 2006 and 2010, in the municipalities that implemented JOGG after 2010 and those who never implemented JOGG, according to:

$$y_{ijt} = \beta_0 + b_{0j} + \beta_1 time + \beta_2 JOGG_{ijf} + \beta_3 time * JOGG_{ijf} + \epsilon_{ij}$$

where $JOGG_{ijf} = 1$ if municipality $j$ in which individual $i$ lives introduces JOGG in the future $f$, sometime after 2010, and $JOGG_{ijf} = 0$ otherwise.

Similar analyses were performed using generalized linear mixed models with the logit link to estimate the effect of JOGG on adherence to fruit/vegetable guidelines over the time.
