# Supplementary material for: Evaluating the contribution of a scaled up community-based overweight prevention programme in the Netherlands to children’s health behaviours and BMIz
Source: Int J Behav Nutr Phys Act. 2025 Jun 18;22:79. doi: 10.1186/s12966-025-01784-x (PMC12177978; doi:10.1186/s12966-025-01784-x)
Supplement: Supplementary file 3 — Supplementary Material 3: Distribution of covariates in non-JOGG and (future) JOGG group over time. [file 12966_2025_1784_MOESM3_ESM.pdf]

Additional file 3 distribution of covariates in non-JOGG and (future) JOGG group over time

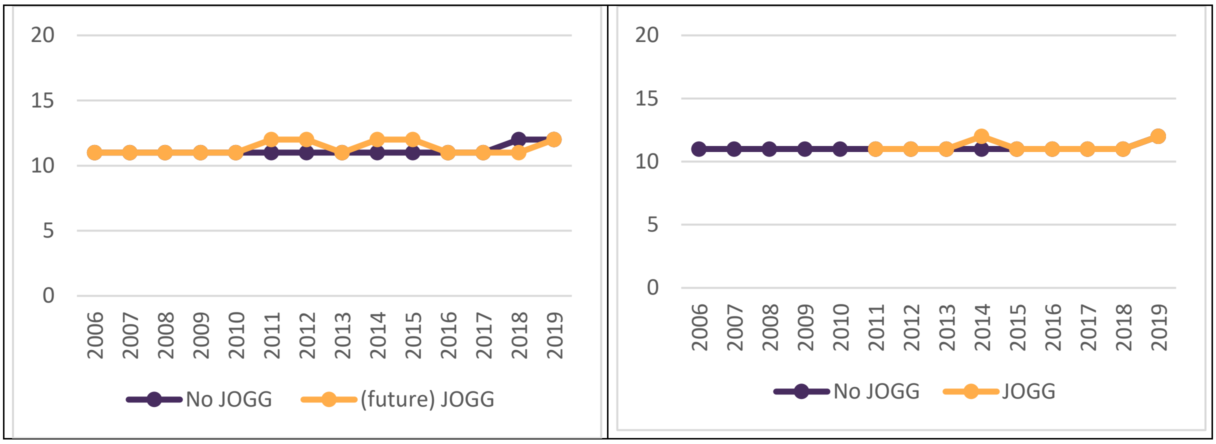

Figure 2A. *Mean age of children*

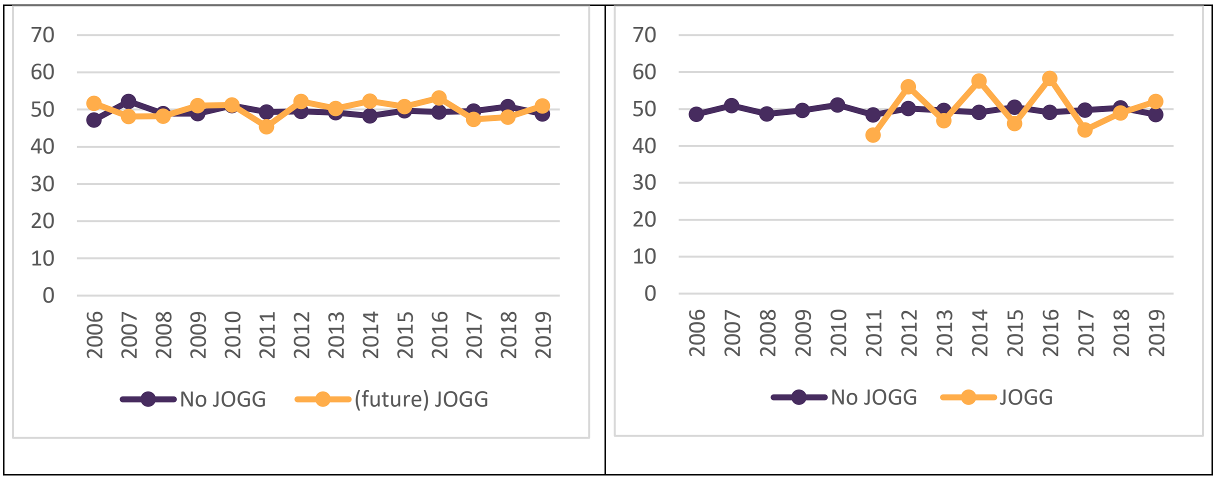

Figure 2B. *Percentage of girls*

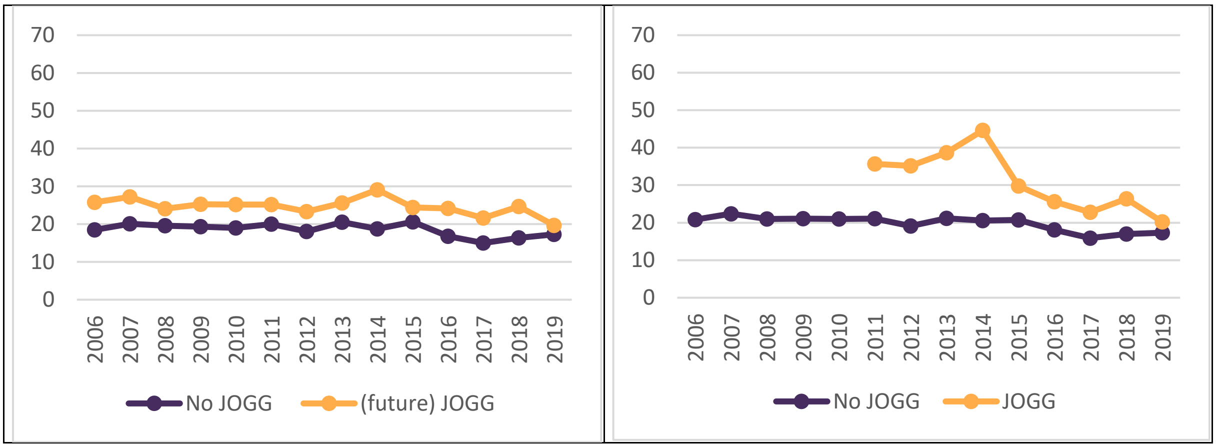

Figure 2C. *Percentage of children from the lowest income quartile*

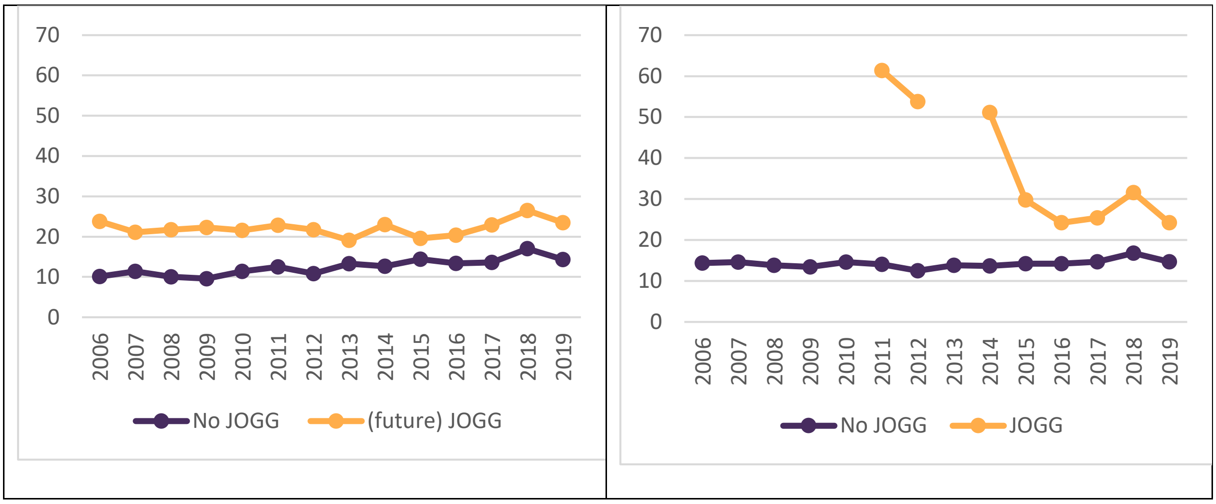

Figure 2D. *Percentage of children with a non-western migration background*

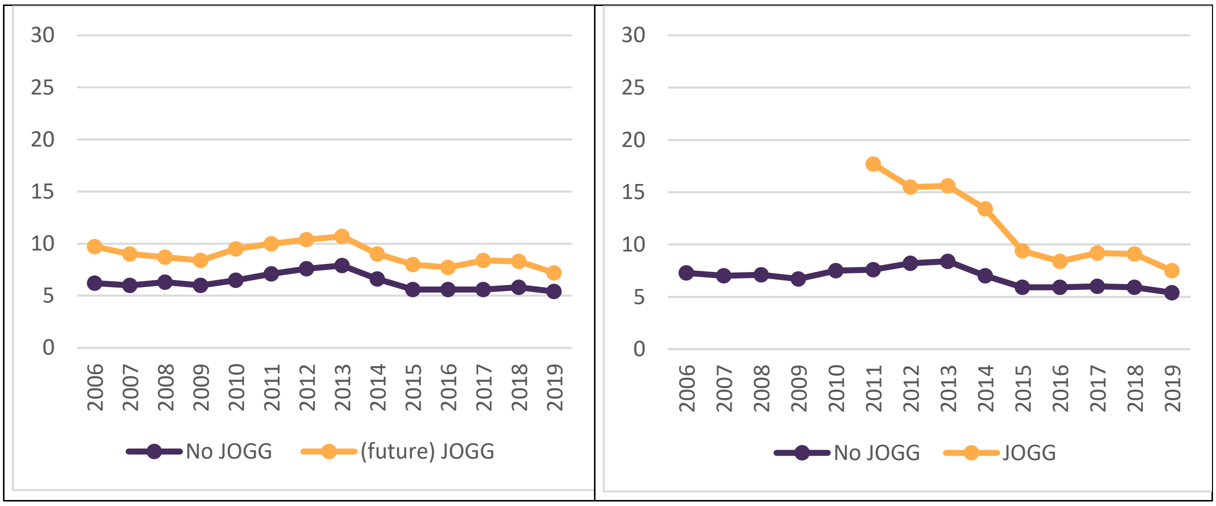

Figure 2E. *Mean neighbourhood deprivation*
