# Supplementary material for: Evaluating the contribution of a scaled up community-based overweight prevention programme in the Netherlands to children’s health behaviours and BMIz
Source: Int J Behav Nutr Phys Act. 2025 Jun 18;22:79. doi: 10.1186/s12966-025-01784-x (PMC12177978; doi:10.1186/s12966-025-01784-x)

Additional file 4 Risk Differences

Figure 3. difference in the probability of adhering to fruit- and/or vegetable guidelines for children exposed to JOGG for at least 18 months compared to no exposure (inc. 95% CI)

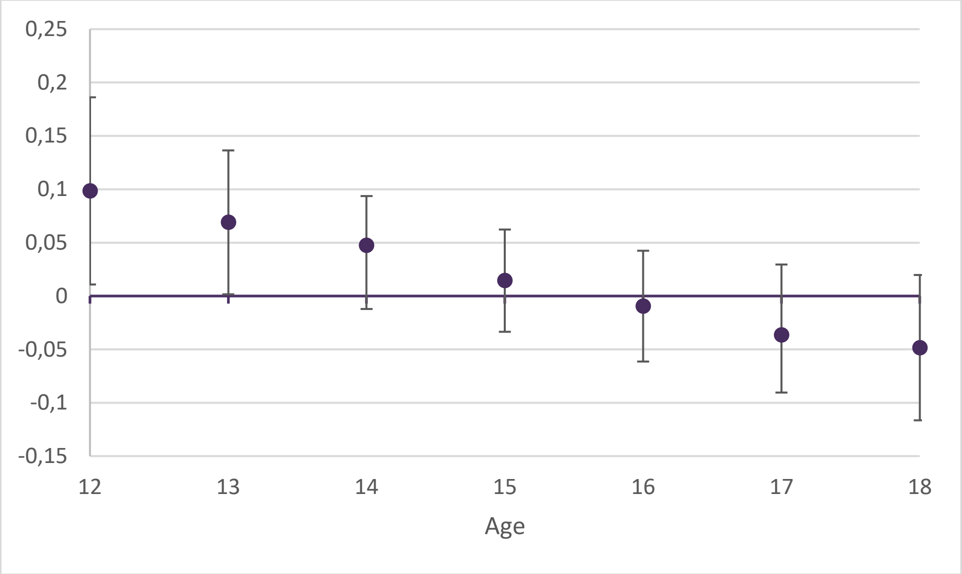

Supplement: Supplementary file 4 — Supplementary Material 4: Risk Differences. [file 12966_2025_1784_MOESM4_ESM.pdf]
