# Supplementary material for: Evaluating the contribution of a scaled up community-based overweight prevention programme in the Netherlands to children’s health behaviours and BMIz
Source: Int J Behav Nutr Phys Act. 2025 Jun 18;22:79. doi: 10.1186/s12966-025-01784-x (PMC12177978; doi:10.1186/s12966-025-01784-x)
Supplement: Supplementary file 5 — Supplementary Material 5: stratified analysis for different introduction years. [file 12966_2025_1784_MOESM5_ESM.pdf]

Additional file 5 stratified analysis for different introduction years

Table 1. Estimates for the difference in intervention outcome trends between JOGG and non-JOGG municipalities for BMIz

Introduction year	Interaction parameter estimates	
	**Beta (95% CI)**	**p-value**
2010-2011	-.02 (-.05, 02)	.30
2012-2013	.02 (-.06, .10)	.60
2014-2015	.04 (-.04, .11)	.35
2016-2017	.15 (-.11, .40)	.26
2018-2019	-	

All models were adjusted for the random effect of municipality

Table 2. Estimates for the difference in intervention outcome trends between JOGG and non-JOGG municipalities for logPA

Introduction year	Interaction parameter estimates	
	**Beta (95% CI)**	**p-value**
2010-2011	-.01 (-.06, .04)	.69
2012-2013	.09 (.00, .18)	.04
2014-2015	.00 (-.08, .08)	.94
2016-2017	.04 (-.20, .29)	.73
2018-2019	-	

All models were adjusted for the random effect of municipality

Table 3. Estimates for the difference in intervention outcome trends between JOGG and non-JOGG municipalities for adherence to fruit-/vegetable guidelines

Introduction year	Interaction parameter estimates	
	**Log-OR (95% CI)**	**p-value**
2010-2011	0.1 (-.12, .13)	.91
2012-2013	-.07 (-.24, .10)	.41
2014-2015	.09 (-.05, .22)	.20
2016-2017	.03 (-.40, .46)	.88
2018-2019	-	

All models were adjusted for the random effect of municipality

[A] log odds < 0 indicate a negative effect on the outcome >0 indicate a positive effect on the outcome
